# August 2020 Interim EuGMS guidance to prepare European Long-Term Care Facilities for COVID-19

**DOI:** 10.1007/s41999-020-00405-z

**Published:** 2020-11-03

**Authors:** Hubert Blain, Yves Rolland, Jos M. G. A. Schols, Antonio Cherubini, Stéphanie Miot, Desmond O’Neill, Finbarr C. Martin, Olivier Guérin, Gaëtan Gavazzi, Jean Bousquet, Mirko Petrovic, Adam L. Gordon, Athanase Benetos

**Affiliations:** 1 Department of Geriatrics, Centre Antonin Balmes, Pôle de Gérontologie du Centre Hospitalier Universitaire de Montpellier, Montpellier University Hospital, Montpellier University, 39 avenue Charles Flahault, 34295 Montpellier Cedex 5, France; 2 INSERM 1027, Toulouse University, Toulouse, France; 3 Department of Health Services Research and Department of Family Medicine, CAPHRI-Maastricht University, Maastricht, The Netherlands; 4 Geriatria, Accettazione geriatrica e Centro di ricerca per l’invecchiamento, IRCCS INRCA, Ancona, Italy; 5 CESP, INSERM U1178, Centre de recherche en Epidemiologie et Santé des Populations, Paris, France; 6 Trinity College Dublin Centre for Health Sciences, Tallaght University Hospital, Dublin, Ireland; 7 Population Health Sciences I King’s College London, London, England; 8 Department of Geriatric Medicine, CHU Nice, University of Nice Sophia Antipolis, Nice, France; 9 Department of Geriatric Medicine, University Hospital of Grenoble-Alpes, Grenoble, France; 10 GREPI TIMC-IMAG CNRS UMR5525, University of Grenoble-Alpes, Grenoble, France; 11 Department of Dermatology and Allergy, Charité, Universitätsmedizin Berlin, Humboldt-Universität Berlin, and Berlin Institute of Health, Comprehensive Allergy Center, Berlin, Germany; 12 MACVIA-France, Montpellier, France; 13 Section of Geriatrics, Department of Internal Medicine and Paediatrics, Ghent University, Ghent, Belgium; 14 Division of Medical Sciences and Graduate Entry Medicine, University of Nottingham, Derby, UK; 15 NIHR Applied Research Collaboration East Midlands (ARC-EM), Nottingham, UK; 16 Department of Geriatrics, CHRU de Nancy and Inserm DCAC, Université de Lorraine, Nancy, France

**Keywords:** COVID-19, SARS-CoV-2, Guidance, Long-term care facility, rRT-PCR, Social isolation

## Abstract

**Purpose:**

The European Geriatric Medicine Society (EuGMS) is launching a second interim guidance whose aim is to prevent the entrance and spread of COVID-19 into long-term care facilities (LTCFs).

**Methods:**

The EuGMS gathered experts to propose a guide of measures to prevent COVID-19 outbreaks in LTCFs. It is based on the specific features of SARS-CoV-2 transmission in LTCFs, residents’ needs, and on experiences conducted in the field.

**Results:**

Asymptomatic COVID-19 residents and staff members contribute substantially to the dissemination of COVID-19 infection in LTCFs. An infection prevention and control focal point should be set up in every LTCF for (1) supervising infection prevention and control measures aimed at keeping COVID-19 out of LTCFs, (2) RT-PCR testing of residents, staff members, and visitors with COVID-19 symptoms, even atypical, and (3) isolating subjects either infected or in contact with infected subjects. When a first LCTF resident or staff member is infected, a facility-wide RT-PCR test–retest strategy should be implemented for detecting all SARS-CoV-2 carriers. Testing should continue until no new COVID-19 cases are identified. The isolation of residents should be limited as much as possible and associated with measures aiming at limiting its negative effects on their mental and somatic health status.

**Conclusions:**

An early recognition of symptoms compatible with COVID-19 may help to diagnose COVID-19 residents and staff more promptly. Subsequently, an earlier testing for SARS-CoV-2 symptomatic and asymptomatic LTCF staff and residents will enable the implementation of appropriate infection prevention and control. The negative effects of social isolation in residents should be limited as much as possible.

## Key summary points

**Aim:**

To guide LTCFs in preventing the entrance and spread of SARS-CoV-2.

**Findings:**

The guidance is based upon the literature available on August 17, 2020. It lists (1) measures that can be implemented to keep COVID-19 out of LTCFs, and (2) COVID-19 symptoms that require RT-PCR testing in residents, staff members and visitors. It also (3) indicates the strategy to be used when a first LCTF resident or staff member is infected, and (4) proposes measures to limit adverse effects of the quarantine of residents tested positive for COVID-19.

**Message:**

The EuGMS guidance enables LTCFs to adapt and suitably implement infection prevention and control measures, considering that the priorities are (1) early detection of symptomatic and asymptomatic COVID-19 residents, staff members and visitors who contribute to the entrance and dissemination of COVID-19 infection in LTCFs and (2) to limit the negative effects of isolation in infected residents.

## Introduction

Long-term care facilities (LTCFs) across Europe encompass a broad range of institution types from home-like facilities to those providing specialised medical care. These facilities include nursing homes, skilled nursing facilities, retirement homes, assisted-living facilities, residential care homes, and palliative care or rehabilitation centres. All these services take care of older people who are no longer able to live independently in the community due to a combination of physical, mental, intellectual or sensory impairments. The European Centre for Disease Prevention and Control estimates that in 2016–2017, there were 64,471 nursing homes, rehabilitation centres and mixed LTCFs in EU/EEA countries, representing a total of 3,440,071 beds [1].

As with the common cold, influenza and other commonly circulating human coronaviruses, LTCFs are high risk environments when it comes to severe outbreaks of SARS-CoV-2 or coronavirus disease (COVID-19). Not only are LTCFs enclosed environments in which the virus can spread rapidly but, more importantly, LTCF residents are extremely vulnerable to complications from the virus, with a mortality rate of > 25% [2, 3].

Coronavirus outbreaks in LTCFs will probably continue to occur in Europe with the likelihood of tens of thousands of additional deaths if:

- Traditional infection-control and public health strategies are not adapted and implemented suitably for the specific features of COVID-19 transmission in LTCFs;
- The diagnostic strategy in residents and staff members is not adapted to the strengths and limitations of the SARS-CoV-2 reverse transcription polymerase chain reaction (RT-PCR) testing;
- No measures are implemented to reduce the mental and physical adverse effects of quarantine in residents when it is necessary.

Taking into account the fact that countries have developed different approaches to prevent COVID-19 epidemics in LTCFs (https://www.jnursinghomeresearch.com/current-issue.html), the specific features of COVID-19 transmission in LTCFs (“Specific features of COVID-19 transmission in LTCFs”), properties of the SARS-CoV-2 testing (“Properties of the SARS-CoV-2 reverse transcription polymerase chain reaction (RT-PCR) testing when implemented in residents and staff members”), and adverse effects of social isolation and quarantine in residents (“Adverse effects of social isolation and quarantine in residents”), the EuGMS is launching a second interim [4] guidance with a checklist aiming at the following: Preventing the entrance of COVID-19 into LTCFs (“Measures to prevent the entrance of COVID-19 into LTCFs by LTCF staff, visitors, and residents, depending on the level of SARS-CoV-2 transmission in the community nearby the LTCF [5] (Box 1)”), reducing the risk of COVID-19 spread within LTCFs (“Measures to prevent the risk of COVID-19 spread by LTCF staff, visitors, and residents, depending on the level of SARS-CoV-2 transmission in the community nearby the LTCF [5] (Box 1)”), limiting the outbreak and optimizing residents’ care when a first resident, staff member, or visitor is infected (“Measures to implement when a new case of COVID-19 is confirmed in a LTCF resident, staff member, or visitor” and “Palliative care for COVID-19 nursing home residents”), educating and supporting staff members (“Staff education to meet LTCF needs in response to the COVID-19 pandemic” and “Staff support to meet LTCF needs in response to the COVID-19 pandemic”).

The current interim EuGMS guidance that is aimed at LTCFs situated in areas with low to substantial community transmission (see Box 1) specifies also policies and waivers (“Policies and waivers to meet LTCF needs in response to the COVID-19 pandemic”) and LTCF organization and structure (“COVID-19 and LTCF organization and structure”) that may help LTCF to meet needs in response to the COVID-19 pandemic.

**Box 1:**
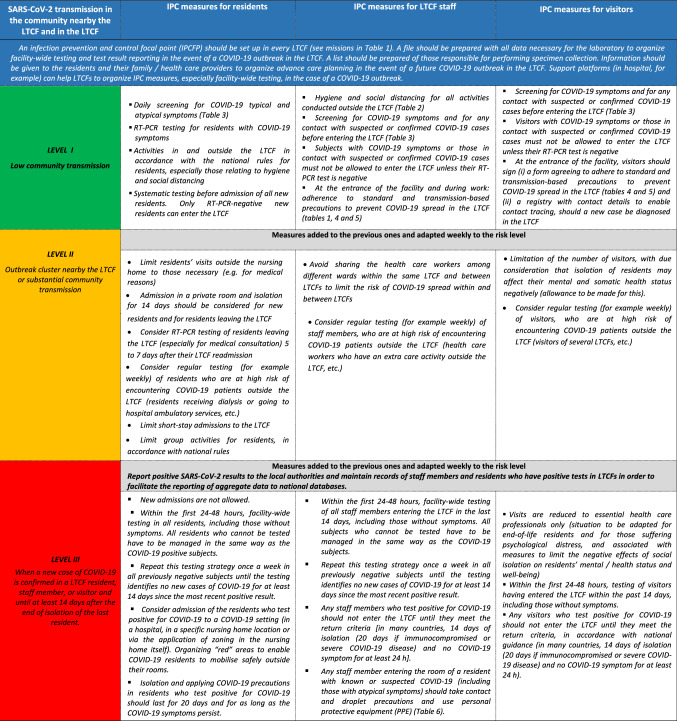
Adaptation of Infection Prevention and Control Measures to SARS-CoV-2 Transmission Level in the Community nearby the LTCF or in the LTCF

The guidance presented here has been formulated by authors from different European countries who have experience with outbreaks of COVID-19 in LTCFs and who are board members of European national geriatric societies or of the EuGMS. The guidance included in this report is based on current knowledge at the time of manuscript transmission (August 25, 2020) (literature review through PubMed and international reports and statements) and will be regularly modified according to newly published data and new experiences. The present guidance, that should not take precedence over local, regional, or national recommendations, should be considered as an aid document for LTCFs to define, depending on local possibilities, the best protection strategy for residents and caregivers and possibly to help write local, regional, or national recommendations when they do not exist. Considering that certain proposed measures (testing, social isolation and quarantine) may have potential adverse effects, the document provides a list of measures that each require an assessment of the risk–benefit ratio on a case-by-case basis. This article also prepares LTCFs for future waves of the pandemic, as the number of COVID-19 cases rises sharply in many countries at the time of manuscript transmission.

## Specific features of COVID-19 transmission in LTCFs

SARS-CoV-2 is highly contagious, due to elevated viral loads in the upper respiratory tract. The long incubation period for COVID-19 (up to 14 days) [5] associated with the fact that asymptomatic residents, visitors, and staff members [3, 6, 7] and residents with atypical COVID-19 symptoms [8–10] contribute substantially to transmission complicates the early detection of cases.

- This suggests that infection prevention and control (IPC) measures aimed at keeping COVID-19 out of LTCFs and early detection of SARS-CoV-2 carriers, including those who are asymptomatic and with atypical symptoms, are essential for limiting transmission in LTCFs [11].
- Family, visiting health care professionals, and LTCF staff initially contributed to the transmission of SARS-CoV-2 within LTCFs, leading to a “lockdown” within the facilities. LTCF staff—including interim temporary or agency staff (hired from private organisations to compensate for gaps in LTCF rotas due to COVID) who moved between LTCFs—were then vectors for virus transmission within the facility and spread among the community [2].
- This suggests that, as part of the reopening process, and for as long as the virus circulates outside the LTCFs, any SARS-CoV-2 introduction and transmission within LTCFs will be due to staff members, visitors, or residents who may have had contact with COVID-19 subjects outside the LTCF, especially during medical examination, hospitalisation or when residents go home or go to their family’s home for visits [11].

## Properties of the SARS-CoV-2 reverse transcription polymerase chain reaction (RT-PCR) testing when implemented in residents and staff members

The reverse transcription polymerase chain reaction (RT-PCR) test, performed using nasopharyngeal swab (or other upper respiratory tract specimens, including throat swab or, more recently, saliva) is currently the most commonly used and reliable test for diagnosing COVID-19 [12]. The United States Centers for Disease Control and Prevention (US CDC) have published useful guidance for Collecting, Handling, and Testing Clinical Specimens for COVID-19 [13]. Quick tests may have low sensitivity compared to the conventional real-time RT-PCR [14]. The RT-PCR test does, however, have some limitations [6, 12, 14], as displayed in Box 2.

**Box 2:**
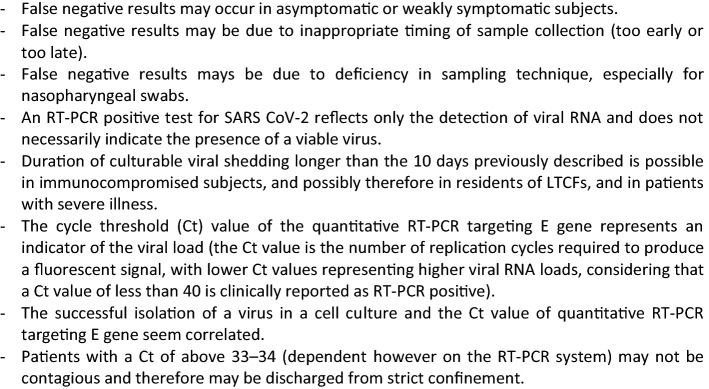
RT-PCR test properties useful when interpreting testing results in LTCF residents, staff members, or visitors [5, 12, 14, 15, 17, 18, 60]

### Sensitivity of SARS-CoV-2 RT-PCR

The sensitivity of the RT-PCR test seems acceptable in LTCF residents, probably higher than 90% (i.e. less than 10% of residents who develop SARS-CoV-2 antibodies in the following days have a negative RT-PCR) [15]. Conversely, RT-PCR testing sensitivity may be lower in LTCF staff members than in residents (meaning that infected staff members may more often test negative by RT-PCR than infected residents) [15]. This suggests that:

- Testing (when possible) residents, staff members, and visitors with COVID-19 symptoms may be an appropriate strategy for the early diagnosis of the first COVID-19 cases in LTCFs [11].
- When a new case of COVID-19 is confirmed in a LTCF resident, staff member, or visitor having entered the LTCF over the past 14 days, LTCF-wide testing (when possible) of all residents and staff members (even asymptomatic) and repeating this testing strategy once a week in all previously negative subjects until the testing identifies no new COVID-19 cases for at least 14 days may be an appropriate strategy for diagnosing asymptomatic SARS-Cov-2 carriers and for preventing SARS-Cov-2 dissemination [11].
- Because RT-PCR testing sensitivity is lower in staff members than in residents (partly because they are less immunocompromised and therefore less often symptomatic than residents when infected) [12], general IPC measures in staff members—including facemasks over the mouth and nose to contain respiratory secretions, hand hygiene and social distancing—is necessary to reduce the risk of transmission of SARS CoV-2 by staff members, even in the case of negative RT-PCR testing [14–16].

### Duration of positive SARS-CoV-2 test results

The duration of the positive SARS-CoV-2 test result may be higher in immunocompromised than in non-immunocompromised subjects (positive RT-PCR testing lasts longer in residents than in staff members), and in subjects with severe disease [12]. Thus, around half of all nursing home residents still have positive test results 14 days after the initial positive results [15].

However, detection of viral RNA 10 days after positive testing in non-immunocompromised asymptomatic subjects and 20 days after positive testing in immunocompromised asymptomatic subjects does not indicate the presence of a viable virus [5, 17]. After that period, most asymptomatic subjects who continue to have detectable SARS-CoV-2 RNA are no longer infectious [17, 18].

- This suggests the need to adapt the duration of isolation and the precautions implemented in subjects tested positive for COVID-19 to their health status (longer in immunocompromised subjects, such as residents, than in non-immunocompromised subjects, such as staff members) and to their symptoms (longer in the case of severe disease or of remaining symptoms) [11, 12, 19].

A few cases have also been reported positive after an initially negative RT-PCR test result. It is unclear as to whether this represents testing error, reinfection, or reactivation [12].

- This suggests that testing may be warranted in subjects who remain or become symptomatic after the above isolation period (10 or 20 days), and in whom an evaluation fails to identify a diagnosis other than SARS-CoV-2 infection [11, 12, 19].

## Adverse effects of social isolation and quarantine in residents

Many older adults in LTCFs undergoing strict social isolation have developed depression, anxiety, worsening dementia, sarcopenia, functional impairment, and failure to thrive.

- This suggests that social isolation has to be restricted to infected subjects and to those in close contact with infected subjects. When necessary, isolation has to be associated as much as possible with measures to prevent negative effects on residents’ mental and health status [20–24].

## Second interim EuGMS guidance to prepare European long-term care facilities for COVID-19

The EuGMS guidance supports the IPC World Health Organization guidance [25] and recommends that an infection prevention and control focal point (IPCFP) should be set up in every LTCF for supervising measures (1) to prevent the entrance of COVID-19 and COVID-19 spread, especially when a new case of COVID-19 is confirmed, (2) to optimize care for infected residents, including palliative care if needed, (3) to educate and support staff members, considering that policies should be adapted to give LTCF providers and professionals flexibility to meet the demands of residents’ care needs in response to the COVID-19 pandemic. To be easily readable tables and boxes have been prepared to be complementary to the text and measures were not detailed when a reference is available.

### Measures to prevent the entrance of COVID-19 into LTCFs by LTCF staff, visitors, and residents, depending on the level of SARS-CoV-2 transmission in the community nearby the LTCF [11] (Box 1)

Box 3 displays IPCFP missions to prevent the entrance of COVID-19 into the LTCF, in accordance with the IPC World Health Organization guidance [25]. The main missions include the following tasks:

- Limitation of the number of visitors, with due consideration that the isolation of residents may have a negative effect on their mental and somatic health status (allowance should be made for this). Indeed, the need to protect residents from the spread of COVID-19 should be balanced with the critical need of support provided by families and other persons [20, 26–29]. LTCF lockdown should therefore be restricted to LTCFs with COVID-19 positive cases or, temporarily, when SARS-CoV-2 circulates strongly outside the LTCF, according to national or local rules.
- General measures in accordance with national rules for residents, staff members, and visitors, especially those relating to hygiene and social distancing, for all activities conducted outside the LTCF (Boxes 4, 5) [19, 30–32].
- Screening for COVID-19 symptoms (the screener should be wearing a surgical mask) before allowing staff members, visitors, or residents to enter the facility (Table 1) [11, 25]. Any contact with suspected or confirmed COVID-19 cases also needs to be checked. Subjects with COVID-19 symptoms or those in contact with suspected or confirmed COVID-19 cases should not be allowed to enter the LTCF unless their RT-PCR test is negative [11].
- Systematic testing before admission of new residents. Only RT-PCR-negative residents can be admitted if the LTCF is free of COVID-19 cases [11].
- Consider testing the residents who have spent a period of time outside the LTCF (at hospital for example), especially when the virus is circulating substantially in the visited area [11].
- Consider regular testing (for example weekly in areas with substantial community transmission) of staff members, visitors, and residents who are at a high risk of encountering COVID-19 outside the LTCF (health care workers who have an extra care activity outside the LTCF, visitors of several LTCFs, residents receiving dialysis or going to hospital ambulatory services, etc.) [11].
- Checking that residents, LTCF staff, and visitors previously tested positive for COVID-19 meet the LTCF entrance criteria according to national guidance. For example, in the US, the UK, and France, return-to-work criteria for staff members with mild to moderate illness who are not severely immunocompromised are: (1) at least 10 days have passed since symptoms first appeared and (2) at least 24 h have passed since last fever without the use of fever-reducing medications and (3) symptoms (e.g., cough, shortness of breath) have improved. In subjects who were asymptomatic throughout their infection: at least 10 days have passed since the date of their first positive viral diagnostic test. In immunocompromised subjects or in subjects with severe illness (most of the residents are immunocompromised, and possibly some staff members and visitors), isolation should last for at least 14 to 20 days and for as long as the subject has symptoms compatible with COVID-19 infection. In subjects who become symptomatic during the 90 days following positive testing and whose evaluation fails to identify a diagnosis other than SARS-CoV-2 infection (e.g., influenza), evaluation for SARS-CoV-2 reinfection is warranted [11].
- Limit short-stay admissions to LTCFs (respite care, rehabilitation, transitional care, as well as intermediate-care, step-down, transitional care, convalescence), unless these residents—who represent a higher infection risk to residents—are in a building or unit with separate entrance, staff and services [26].

**Box 3:**
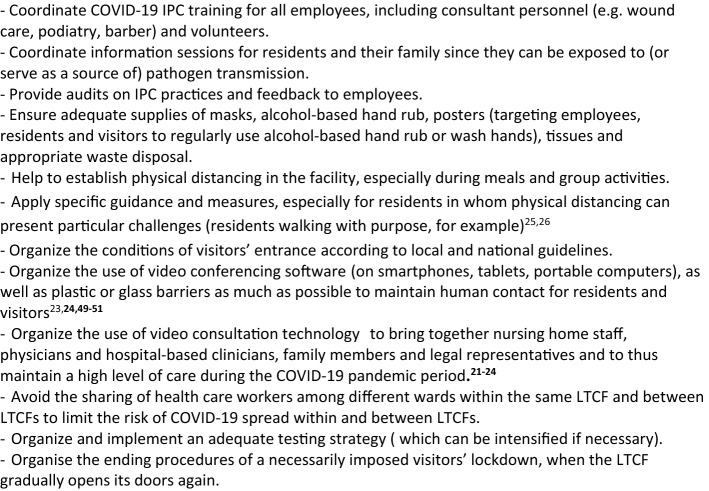
Tasks of the Infection Prevention Control (IPC) focal point in LTCFs

**Box 4:**
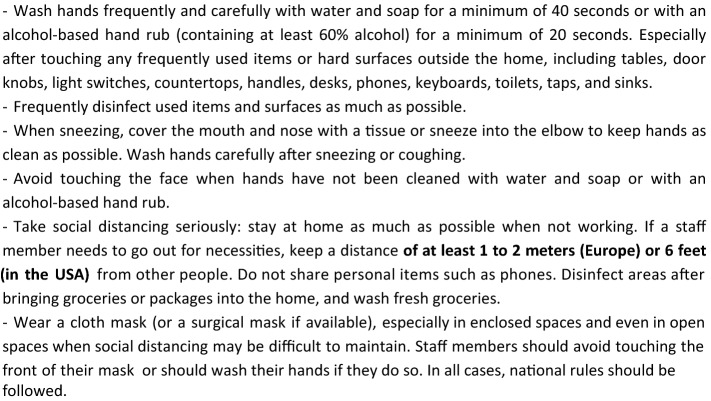
Recommended hygiene and distancing measures in the daily life of the health care personnel, visitors, and residents who go outside the LTCF, for as long as the virus is circulating outside the LTCF

**Box 5:**
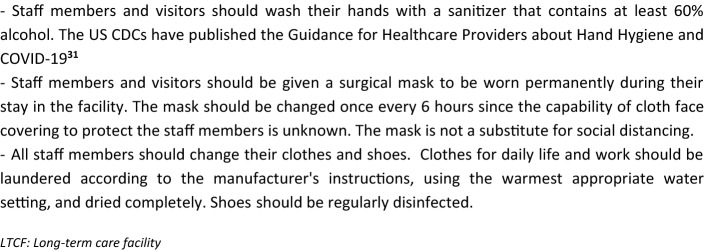
Measures that should be taken by staff members, visitors, and residents before they enter the facility, whether COVID-19 positive or negative, for as long as the virus is circulating outside the LTCF, especially in areas with substantial community transmission

**Table 1:** Symptoms compatible with COVID-19 in the general population and in frail older people

Typical COVID-19 symptoms in the general population and in frail older people	Atypical COVID-19 symptoms possibly observed in frail older people
Temperature ≥ 37.5 °C	Transient episodes of fever
Cough	Oscillating temperature with transient episodes of hypothermia
Fatigue	Malaise
Sputum	Transient episodes of hypotension, with mottling
Shortness of breath	Oxygen desaturation (< 95%), increased respiratory rate (> 25/min)
Muscle aches	
Sore throat	Anorexia, dry mouth, loss of weight
Headache	Delirium, apathy, disorientation, lethargy, somnolence
Anosmia	Balance impairment, dizziness, falls
Chills	
Nasal congestion	Conjunctivitis
Nausea or vomiting	Diarrhoea

### Measures to prevent the risk of COVID-19 spread by LTCF staff, visitors, and residents, depending on the level of SARS-CoV-2 transmission in the community nearby the LTCF [11] (Box 1)

The infection prevention and control focal point (IPCFP) should also organize the following tasks:

- Healthcare professionals and family members visiting the LTCF and allowed to enter the facilities should sign a form agreeing to adhere to standard and transmission-based precautions to prevent a COVID-19 spread within the LTCF (Boxes 5, 6). They should also sign a registry with contact details to facilitate testing and contact tracing should a new case be diagnosed in the LTCF. Face masks, hand hygiene and social distancing are consensual measures when entering a European LTCF, and masks should be available at the LTCF front entrance. Some countries, such as the UK, apply stricter measures, with staff members wearing gloves and aprons for any form of resident care, and all visitors required to don the same Personal Protective Equipment as a condition of entry.

**Box 6:**
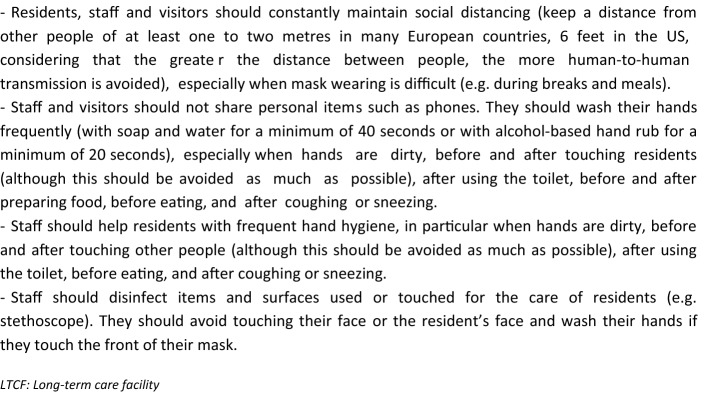
Measures that should be taken by staff members, visitors, and residents during their stay in the LTCF for as long as the virus is circulating outside the LTCF, especially in areas with substantial community transmission
- The US CDC has published specific guidance for standard and transmission-based precautions to prevent COVID-19 spread in the memory care units of LTCFs [33].
- Every new resident should be admitted to a single occupant room and isolated for 14 days, in the same way as residents leaving the LTCF (for a medical examination or to visit families for example). If COVID-19 symptoms occur (Table 1), the resident should be tested. Due to the fact that COVID-19 residents can be asymptomatic, those leaving the LTCF (especially for medical consultation) should be ideally tested 5 to 7 days after their LTCF return in areas with substantial community transmission.
- Daily screening for COVID-19 symptoms (including atypical symptoms) (Box 2) should be incorporated into the routine daily observation with clear COVID alerting features. Testing should take place if there is any suspicion of infection. LTCFs should be provided with a sufficient supply of test kits for PCR testing to meet diagnostic needs, with access to a laboratory that can provide results of PCR testing within 24 h if possible [34].
- Administrative records should be updated daily on the basis of COVID-19 symptoms, to record whether a resident has received a COVID-19 test, has been isolated due to COVID-compatible symptoms, and/or has required any other non-standard IPC measures.

The IPCFP should also:

- Provide information to the residents and their family/health care providers and organize advance care planning to support them in understanding and sharing their personal values, life goals, and preferences regarding medical care, including appropriate advance directives (ADs) and decisions about escalation to hospital in the event of any future COVID-19 outbreak in the LTCF. An explanation of the limited success of mechanical ventilation in older adults with COVID-19 should be given as well as a description of care that can be provided in the LTCF in the event of non-hospitalization [35]. Advance care planning has to be timely and proactively executed to provide high-quality long-term care, contributing to the quality of life in the remaining life span of the residents [35].
- Prepare a file with all data necessary for the laboratory to organize testing and test result reporting (name, address, date of birth, insurance data, etc. of all residents, staff members, and visitors) in the event of a COVID-19 outbreak in the LTCF. There should also be a list of those responsible for performing specimen collection, including the facility’s trained staff members. These people need to be trained to collect specimens correctly, using the appropriate personal protective equipment (PPE) [36].

In a cohort study of French LTCFs, mortality rates related to COVID-19 were lower among nursing homes that implemented staff confinement with residents compared with those in a national survey, suggesting that self-confinement of staff members with residents in areas with substantial community transmission may help protect LTCF residents from mortality related to COVID-19 and residents and staff from COVID-19 infection [37].

### Measures to implement when a new case of COVID-19 is confirmed in a LTCF resident, staff member, or visitor

In some countries, COVID-19 geriatric support platforms have been set up in local hospitals, often in conjunction with public health, with a hotline for LTCFs [38]. These support platforms have been shown to be helpful for LTCFs in organizing IPC measures, especially repeated facility-wide RT-PCR testing and reporting of test results. The hotline can be useful for responding to the information requirements of the LTCF staff regarding care procedures, in connection with other specialized units, (emergency, intensive care, palliative care, pulmonary medicine, infectious diseases and hygiene units), possibly by telemedicine [39].

Measures that need to be taken as quickly as possible when a new case of COVID-19 is confirmed in a resident, staff member, or visitor include the following:

Within the first 12 h:

- Staff should be made aware of the existence of the advance care planning, including advance preferences or directives for all residents who provide consent [35].
- Residents should be restricted to their room and visits reduced to essential health care professionals only (situation to be adapted for end-of-life residents and for those suffering psychological distress, and associated with measures to limit the negative effects of social isolation on residents’ mental/health status and well-being) [19, 20, 26] (Box 3). Any staff members or visitors who test positive for COVID-19 should not enter the LTCF until they meet the return criteria, in accordance with national guidance (see “Measures to prevent the entrance of COVID-19 into LTCFs by LTCF staff, visitors, and residents, depending on the level of SARS-CoV-2 transmission in the community nearby the LTCF [5] (Box 1)”) [17].
- If it is not possible to exclude staff members for organizational reasons, the US CDC has published Strategies to Mitigate Healthcare Personnel Staffing Shortages [40].
- If a resident tests positive for COVID-19, admission to a COVID-19 setting specialized in geriatric care (in hospital, in a specific nursing home location if such facilities are available with, if appropriate, agreement of family/caregiver/legal representative) should be considered or set up via the application of zoning in the nursing home itself. This will separate the infected resident from the others, and optimize care for the COVID-19 residents [11, 15, 34, 41]. Excluding all new positive residents during each facility-wide testing may reduce the LTCF staff workload, optimize IPCs and reduce the LTCF “viral load”, therefore reducing COVID-19 spread in the LTCF. If the LTCF can provide appropriate isolation and staffing, an area of the facility should be dedicated for the care of residents with suspected or confirmed COVID-19. There should be a specific staff and a clear delineation of risk zones [41]. Organized staff into teams who work exclusively within COVID-19 and non-COVID-19 zones is strongly recommended [38, 42]. Because working exclusively with COVID-19 residents is physically and emotionally exhausting, thought should be given as to how to rotate staff teams, allowing appropriate periods of leave (at least 7 days), between working in COVID-19 units and returning to the mainstream. Distinction should also be made between residents able to understand/tolerate their isolation (e.g. cognitively intact residents) and those more likely to suffer from isolation (e.g. residents with dementia and depressed mood or who walk with purpose) who may be better cared for in cohorted or zoned areas. LTCF COVID-19 units should receive transfers from within the facility as well as new admissions/re-admissions from COVID-19-positive hospitals. In Lombardy (Italy) however, public policies aimed at discharging COVID-19 cases from hospitals to LTCFs to improve the capacity of the health systems to face the lack of hospital beds are suspected to have also increased the risk of infection in the nursing homes’ hosts [43, 44].

Therefore, if possible, such a COVID-19 LTCF unit should have a separate entrance/exit or try to install temporary walls or doorways at the entrance. If it is not possible to segregate COVID-19-positive patients in a separate unit, such patients should be cohorted in one area of the affected unit and dedicated staff should be assigned to care for them. This is particularly important for residents with dementia [45].

- The establishment of COVID-positive “red” areas also enables residents, with and without dementia, to mobilise safely outside their rooms, minimising the risk of deconditioning associated with prolonged isolation in a private room [41, 42].

Any staff member entering the room of a resident with known or suspected COVID-19 (including those with atypical symptoms) should adhere to standard precautions [45], apply contact and droplet precautions and use personal protective equipment (PPE) (Box 7).

**Box 7:**
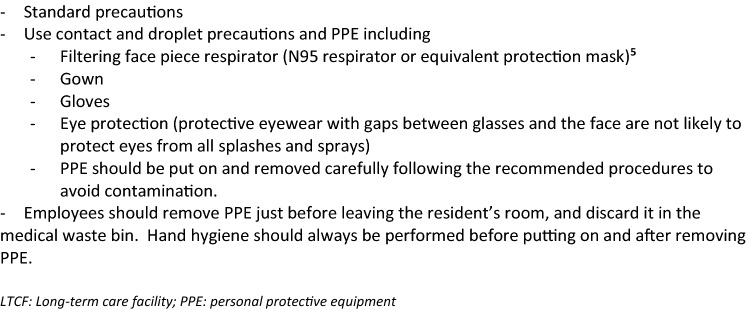
Precautions for LTCF staff who enter the room of a patient with known or suspected COVID-19 [25, 30, 32, 44]

Within the first 24–48 h:

- Facility-wide testing of all LTCF residents, staff members, and visitors having entered the LTCF over the past 14 days, including those without symptoms. This can be challenging for some of the residents, especially with cognitive impairment, for staff members, and for visitors who may refuse testing. All subjects who cannot be tested have to be managed in the same way as COVID-19 positive subjects  [45].
- Repeat this testing strategy once a week in all previously negative subjects until the testing identifies no new COVID-19 cases for at least 14 days since the most recent positive result. The US CDC has published guidance for preparing LTCFs to perform facility-wide testing [46]. The LTCFs should be prioritized for rapid, point of care testing as this is the best way to manage the epidemic in real time [34].
- Report positive SARS-CoV-2 results to local authorities and maintain records of staff members and residents who have positive tests to facilitate the reporting of aggregate data to national databases [11].
- Assess IPC practices with help from the proximity COVID-19 support platform for LTCFs or a hygiene platform in the proximity hospital while conducting facility-wide testing [47].
- Isolation and COVID-19 precautions in residents tested positive for COVID-19 should last until they meet the end of isolation criteria, in accordance with national guidance. As indicated previously, isolation and COVID-19 related precautions in residents/staff members previously tested positive for COVID-19 should probably end safely [17]:

- In non-immunocompromised persons: 10 days after symptom onset (or 10 days after the positive testing in subjects who are not symptomatic) and when subjects are asymptomatic for at least 24 h.
- In immunocompromised persons (the case for most of the LTCF residents) or those with a severe illness: 20 days after symptom onset or after positive testing and when subjects are asymptomatic for at least 24 h. Some experts recommend to end isolation 14 days after positive testing and when subjects have been asymptomatic for at least 24 h [34].
- If control testing is implemented in patients with a Ct of above 33 (see definition in Box 2) [18]:
  - Entries are only possible at least 14 days after the end of the isolation of the last resident tested positive when no new residents and staff members have any signs of COVID-19 [11].
  - Because immobility may lead to sarcopenia and functional impairment [29], measures should also be implemented to limit the physical effects of quarantine in residents [48], associated with measures to limit the risk of deep vein thrombosis, COVID-19 being associated with a procoagulant state [34, 49]. One example of physical activity programme that can be proposed in residents who are isolated in their room is the MATCH (Maintenance of Autonomy Through exercise Care during Hospitalization) programme that comprise specific and adapted exercises (e.g., seated knee extension, sit to stand, step aside, chair for-ward bend, bipedal and unipedal static balance, and wall squat) and walking time. This programme, available for notebook with optional and additional training video, has been tested and implemented during this COVID-19 pandemic in geriatric hospital units and long-term care with no individual Internet access [48].

### Palliative care for COVID-19 nursing home residents

As indicated previously, advance care planning has to be timely and proactively discussed with the resident and/or their family, including “do not resuscitate”, “do not intubate”, and “do not hospitalize” [35]. Physicians, nurse practitioners, physician assistants, and social workers can contribute to the discussion. Conversations should include an explanation of the limited success of mechanical ventilation in older adults with COVID-19, and a description of the type of care which can be provided if non-hospitalization is decided. Conversations should emphasise the fact that symptoms of a resident with COVID-19 can worsen rapidly [34].

Proposals for the management of COVID-19 end-of-life symptoms consistent with realistic goals of care, and tailored with respect to comorbidities and symptom burden, are displayed in Table 2. The discontinuation of non-essential medications should be considered (including intravenous fluids that can induce dyspnoea and increase secretions and prophylactic anticoagulation, weighing this against the fact that COVID-19 is procoagulant) to improve resident comfort and reduce nursing administration time. Checking labs or examinations that can expose residents and staff should be avoided [49].

**Table 2:** Management of COVID-19 end-of-life symptoms on-site adapted from ([34])

Symptom	Consider	Tailored on comorbidities and symptom burden
Fever	Acetaminophen	As needed (PRN) or standing doses
Pain	Acetaminophen Morphine Oxycodone	As needed (PRN) or regular doses Start at 2.5 mg or 5 mg PO/SubL q4–6 h: avoid repeated morphine doses if CrCl < 30 20 mg/mL, consider start at 2.5 mg or 5 mg PO (per os)/SubL (sublingual) q4–6 h
	Higher doses of morphine or oxycodone for higher symptom burden and non-opioid naïve patients	
Dyspnea	Oxygen by nasal cannula if capillary pulse oxygen is < 90% Advance to ventimask if hypoxia not improved Limit intravenous fluids Morphine Oxycodone Parenteral benzodiazepine e.g. Lorazepam 2 mg/mL Antibiotics if concern for bacterial pneumonia	Titrate as needed See above See above Start 0.5 mg PO/SubL q6–12 h
Restlessness	Parenteral benzodiazepine	See above
Excess secretions	Risk of delirium with atropine; limit intravenous fluids Atropine eye drops used sublingually Glyclopyrrolate if PO possible Scopolamine patch (anticholinergic side effects)	1 drop q2h 1‒2 mg bid-tid PRN

COVID-19 makes it difficult to hold face-to-face end-of-life discussions and increases the chances of the resident dying without someone present [50]. To avoid situations in which the infected residents die without the possibility to say goodbye to their relatives, LTCFs should organize a separate room in which the infected resident may be placed and should take special precautions to allow families to be present when the loved one passes. In some cases, the resident will have no relatives and an LTCF staff member becomes his/her very close everyday caregiver. In this case, the death of the resident may have a detrimental impact on the mental health of the care assistants/nurses, justifying the need to develop programmes allowing reflection and relief of emotions to reduce the burden for both the relatives and the nursing home staff [51].

### Staff education to meet LTCF needs in response to the COVID-19 pandemic

The COVID pandemic highlights the need for universal adoption of standards of medical care for physicians and other staff members in nursing homes in Europe [52]. Indeed, in this COVID-19 pandemic period, the assessment of patients prior to admission by a specialist in geriatric medicine or old-age psychiatry (or both if necessary) is more useful than ever, to (1) detect and remediate illness and functional loss, and (2) better delineate care needs for the older person, so as to clarify whether NH admission can be avoided or deferred. The multiple missions of IPCFP necessitate a medical leadership (the US medical director can serve as a model) to coordinate the implementation of the above measures. These measure should be set up in collaboration with (1) nurses who have gerontological training, especially in dementia and palliative care, (2) care attendants who have due training in the care of older people, (3) physicians with a formal competencies in geriatric medicine and old-age psychiatry, and (4) specialist gerontology services, including geriatricians, old-age psychiatry and clinical nurse specialists as well as (5) specialists in palliative care support. COVID-19 geriatric support platforms can serve to liaise with IPCFP, hospital geriatric units (public or private), clinical nurse specialists, specialists in palliative care and infectious diseases, IPCFP of other LTCFs in the region, and public health [47]. In a recent study, every 20 min (per resident day) increase in registered nurse staffing was associated with a 22% reduction in the number of confirmed COVID-19 cases (in facilities with at least one confirmed case) and with 26% fewer COVID-19 deaths (in facilities with at least one death due to this infection). This confirms the importance of the qualified staff in preventing SARS-CoV-2 dissemination in LTCFs [53].

### Staff support to meet LTCF needs in response to the COVID-19 pandemic

The chronic understaffing in LTCFs—due to part-time employment, punitive measures related to sick leave, low wages, obligation to work when unwell—explains why it may be difficult in most LTCFs to (1) monitor residents for COVID-19, (2) follow protocols to keep residents physically distant and (3) implement public health measures to reduce the transmission of the virus. This is particularly the case when isolating COVID-19 residents with dementia who may not understand or follow the procedure exacerbates their behaviour [54].

The workload of LTCF staff members is has been increased by the pandemic because of: the ban of family members and other volunteers who provide supportive care; and the increase of staff absenteeism due to COVID-19 symptom and/or fear of contracting the virus or transmitting it to their loved ones. In addition, due to the COVID-19 pandemic, the actions of health authorities have generated a feeling of abandonment in LTCF staff members and of degrading [55]. Coupled with the moral distress of losing residents they have cared for over many years, the potential guilt of their own role in transmission, and the feeling of violating their own ethical or moral codes when working under high pressure, there is a high risk of staff experiencing psychological trauma in LTCFs. Because LTCF staff members need more support and recognition than ever, considerations for nursing home leaders and regulators to support the health and well-being of nursing home staff and residents have been proposed. These include (1) measures to keep staff informed (because expert advice evolves) and (2) health measures (focussing on stress management and meeting the basic needs of staff) [54], by (a) optimizing human resources (redeploying and educating staff from other health care facilities (such as hospitals) to work in nursing homes), (b) implementing new clinical practices related to COVID-19, especially end-of-life care including advanced care planning, symptom relief, and post mortem care, (c) providing education and training, and (d) acquiring PPE [56]. In Hong Kong, for example, all health care institutions keep PPE in stock (exchanged every 3 months) [56].

## Policies and waivers to meet LTCF needs in response to the COVID-19 pandemic

Policies and waivers implemented by the American Centers for Medicare and Medicaid Services (CMS) to give long-term care providers and professionals flexibility to meet the demands of resident and patient care needs in response to the COVID-19 pandemic can be very useful for the European countries [57]. These policies and waivers include the possibility for hospitals to deliver care in temporary expansion sites to accommodate COVID-19 patients, including COVID-19 residents. LTCFs can be repurposed as expansion sites and designated as COVID-19 patient care facilities to achieve population separation and transmission mitigation.

CMS have also enabled the opening or use of non-LTCF buildings for unmet COVID-19 isolation and treatment as well as the temporary use of rooms in the facilities (activity rooms, meeting/conference rooms, or dining rooms) for resident care, to expand surge capacity and for purposes of cohorting and infection control.

As described in Box 3, telehealth provides new opportunities to enhance healthcare quality in LTCFs that include specialty consultations by physicians and non-physician practitioners where appropriate [22].

CMS also allow for physician delegation of tasks or required visits to a physician assistant, nurse practitioner, or clinical nurse specialist. CMS recommend that a LTCF may not employ a nurse assistant for longer than four months unless he/she meets certain training/certification requirements. The CMS allow for the redeployment of employed physicians, utilization of available trainee staff, and recruitment of community and retired physicians in LTCFs.

It must however be strongly reminded that all new HCPs must adhere to standard and transmission-based precautions to prevent a COVID-19 spread within the LTCF (Box 6).

To enable clinicians to better focus on the care needs of residents, CMS have temporarily given LTCF providers relief from paperwork, reporting and audit requirements [57].

## COVID-19 and LTCF organization and structure

The probability of having a COVID-19 case in LTCFs, such as other infections (i*nfluenza*, *Norovirus, Clostridium difficile*), is higher for larger facilities in urban locations [58]. The COVID-19 pandemic offers the opportunity to rethink the design of LTCFs so as to provide a high quality of regulating ventilation and temperature, divide them into smaller wards by clustering smaller numbers of residents in living and/or dining rooms, or even in small-scale freestanding living facilities (such as the Green House Model or Green Care Farms), with more space per resident, separate offices spaces and additional separate rooms for residents who need isolation due to infection, and to provide the possibility for family members to stay with the resident at the time of his/her death [52, 59].

## Conclusion

This second EuGMS Interim guidance to prepare European Long-term care facilities for COVID-19 takes into account studies, guidance, documents, and experience available up to August 25, 2020.

Any changes to the current guidance will be published according to new knowledge on this topic, especially once data are available on the follow-up of symptoms, interventions, course of the disease in COVID-19-positive residents, vaccination schedules, point of care testing, interactions between COVID-19 and flu, and on serology in residents, visitors, and staff members.
